# Co-Operative Appeals

**Published:** 1919-02-01

**Authors:** 


### Co-operative Appeals.

*To the Editor of* The Hospital.

Sir,—I think it was in October last that the Editor of the *Gentlewoman* made an appeal on behalf of six London institutions. In your issue of November 20 last you published a letter under the title "Co-operative Appeals," in which it was said that it was hoped the six hospitals concerned would reap a rich reward, though the writer of the letter seemed to throw out a hint that in his opinion individual effort paid best in appeal matters, and that there was no reason to suppose that the head of the Editor of the *Gentlewoman* was stronger and better than the six heads of the six secretaries of the hospitals on whose behalf the appeal was made.

It would be interesting to the readers of The Hospital if you could ascertain the result to date of "A Costless Thank-offering." Of late I, as a reader of many publications, have not come across a single reference to this "Costless Thank-offering" business, but I trust I am not right in supposing that the matter has ended. The great silence on the subject is surely not a sign that the "Costless Thank-offering" has turned out to be (to use a well-known Americanism) a "priceless frost." Any information that you can give your mystified readers will be warmly appreciated.—Yours faithfully,

January 21, 1919. Another Hospital Secretary.

